# Reviewed article: Pinheiro HA, Cerceu VR, Pinheiro LA, Pagotto V, Menezes RL. Prevalence and factors associated with sarcopenia in elderly people referred by Primary Healthcare to a specialized service in an area of the Federal District of Brazil, 2015-2017. Epidemiol Serv Saude. 2025:34;e20240500.

**DOI:** 10.1590/S2237-96222025v34e20240500.a

**Published:** 2025-08-04

**Authors:** Nathan Aratani

**Affiliations:** Universidade Federal de Mato Grosso do Sul, Instituto Integrado de Saúde

**Table:** 

Rating	Excellent	Good	Average	Below average	Poor
Interest		✓			
Quality				✓	
Originality		✓			
Overall		✓			

**Table:** 

Questionnaire	Yes	No	Not applicable
Does the manuscript contain new and significant information to justify publication?	✓		
Does the Abstract (Summary) clearly and accurately describe the content of the article?	✓		
Is the problem significant and concisely stated?		✓	
Are the methods described comprehensively?		✓	
Are the interpretations and conclusions justified by the results?	✓		
Is adequate reference made to other work in the field?	✓		

## Manuscript Structure

Length of article is: Adequate

Number of tables is: Too few

Number of figures is: Adequate

Please state any conflict(s) of interest that you have in relation to the review of this paper (state “none” if this is not applicable): None

## Comments

A temática do artigo é relevante para o SUS e a prática profissional. Para uma oportuna conclusão dos resultados alguns aspectos necessitam de revisão, a saber:

REVISÕES MAIORES:

1. A pergunta de pesquisa precisa ser mais bem definida. Sugiro suprimir do objetivo o trecho “é uma preocupação para o futuro ou um desafio na atualidade”.

2. A prevalência de sarcopenia é para a população encaminhada pela atenção primária ao serviço especializado, precisa deixar claro no método, o estudo não é representativo de toda a população idosa que foi atendida pela atenção primária.

3. Recomendo revisão da definição do objetivo da pesquisa do resumo e do corpo do texto, que estão diferentes.

4. O delineamento do estudo no resumo está diferente do apresentado no item de métodos. O delineamento realizado foi do tipo de estudo transversal.

5. Recomendo fortemente revisão do texto do resumo com um todo, as ideias podem ter melhor coesão e complementariedade entre elas. Revise o resumo conforme normas aos autores da revista. Apresente objetivo conforme o método e dados analisados (por sexo ou por sarcopenia) e conclusão que o responda a partir dos resultados apresentados no resumo. Descreva quais e como foram calculadas medidas de frequência ou associação e de dispersão.

6. É importante rever as instruções aos autores da revista para a apresentação dos dados do resumo, valores percentuais devem ser apresentados com duas casas decimais após a vírgula.

7. Observar o formulário STROBE para redação do artigo.

8. O delineamento do estudo precisa ser mais adequado à pergunta de pesquisa. Não está claro qual foi o desfecho investigado, se foi de prevalência de sarcopenia entre homens e mulheres (variável dependente), ou se foi desfecho entre pacientes com e sem sarcopenia (variável dependente). Sarcopenia está indicada como variável independente, mas na apresentação dos resultados isso não fica claro.

9. Revise os métodos e resultados para garantir que o delineamento informado condiz com a pesquisa. A depender da perspectiva adota, pode ser que o estudo se enquadre melhor na perspectiva de estudo do tipo caso-controle, ao invés de estudo transversal.

10. Descreva os métodos com mais detalhes, incluindo o processo de coleta e análise de dados, para que outros pesquisadores possam replicar o estudo. Explique nos critérios de inclusão e exclusão quem é a população que é encaminhada pela atenção primária que foi captada pela pesquisa, são pessoas que tem critério de acompanhamento? São pessoas com algum diagnóstico a confirmar? Por qual razão eles foram encaminhados pela atenção primária?

11. Apresentar valor de margem de erro considerada no cálculo amostral.

12. Quando citam o teste cognitivo como critério de inclusão e exclusão, quem aplicou o teste e em qual momento?

13. É importante citar o que está considerando como pessoa idosa, qual faixa etária está sendo considerada?

14. Revise a alocação dos textos e informações do método, há itens que falam sobre as variáveis de interesse do estudo, que estão relatados como critério de inclusão e exclusão da amostra. Exemplo: citação dos aspectos socioeconômicos, comorbidades, atividade física regular e etc.

15. Quando descreve que foram realizadas três medidas de força e teste de marcha, deve ser citado se foi realizado por um único pesquisador ou vários, e se foram treinados previamente para minimizar o viés de avaliação.

16. Os métodos estatísticos aparentam ser inadequados aos dados do estudo e precisam ser corrigidos de acordo com as melhores práticas de análise de dados. Foi aplicado o teste de razão de prevalência (RP), mas a amostra do estudo é de um recorte populacional de um grupo específico (idosos encaminhados pela atenção primária para o ambulatório por algum motivo a ser esclarecido). A análise mais indicada seria teste de Razão de Chances (Odds ratio), que permite calcular associação entre os grupos comparados.

17. As tabelas e figuras precisam ser revisadas para garantir clareza e precisão para compreender qual foi a amostra final e quais foram os grupos caso e controle incluídos na análise estatística.

18. Os títulos das tabelas precisam representar as informações e dados apresentados. Nas tabelas 1 e 2 menciona que irá apresentar “distribuição da amostra, prevalência e fatores associados”, mas não tem os dados da distribuição da amostra e da prevalência, apenas os valores de associação. Recomendo que seja apresentado uma tabela com dados exclusivos da distribuição da amostra (valores absolutos) e prevalência (porcentagem). Uma segunda tabela com os resultados do teste de associação.

19. Para a elaboração e apresentação da tabela sugerimos consultar novamente as orientações aos autores da revista para ajuste na forma de apresentação dos dados. Numerais de p-valor devem ser apresentados com 3 casas decimais após a vírgula, não é indicado fazer o arredondamento (exemplo p-valor: 0,07).

20. A figura 1 é ilustrativa para definição de paciente com sarcopenia ou não, mas tem limitação para indicar qual a população final que compôs a amostra do estudo. 900 usuários foram encaminhados pela APS, 400 atendiam aos critério de exclusão. Dos 500 indivíduos, 138 tinham força de preensão normal e foram excluídos do estudo?

21. Dos 362 indivíduos com provável sarcopenia, 163 tinham circunferência da panturrilha normal, foram excluídos do estudo?

22. Os resultados das tabelas 1 e 2 representam os usuários com desempenho físico >0,8m/s (n=161), assim a amostra do estudo foi dessa população, não dos 362 com provável sarcopenia, ou dos 500 que atenderam aos critérios de inclusão e exclusão.

23. Verifique a pertinência de incluir as seguintes palavras-chaves já existentes no DeCS e que melhor descrevem sua pesquisa: Pessoa idosa; Idoso. Medidas de AssociaçãoExposição-Risco ou Desfecho; Medidas de Associação, Exposição, Risco ou Desfecho

24. Explique como os resultados podem ser generalizados e aplicados em outros contextos, justificando a relevância do estudo para diferentes populações. O resultado apresentado (n=161) não é representativo da população, pois não atinge a amostra estimada no método. A amostra pode também não ser representativa dos usuários da atenção primária, pois só foram incluídos usuários encaminhados pela atenção primária ao ambulatório, por alguma razão que precisa ser relatado.

25. Relate a proporção de perdas de participantes e/ou a taxa de resposta, discutindo o impacto potencial dessas perdas nos resultados do estudo.” A amostra incluída foi inferior a calculada no método.

26. No resultado cita que participaram 500 idosos, mas na tabela de resultados só foram incluídos 161 indivíduos. Os resultados sugerem que participaram da pesquisa 161, se o desfecho de interesse eram mulheres e homens com sarcopenia. Se o desfecho era sarcopenia ou não (variável dependente), ai sim justifica a indicação de participação de 500 indivíduos. Os resultados de associação são representativos das pessoas com sarcopenia (n=161), e não dos com sarcopenia grave (n-38) ou da população idosa (n=500). Rever os métodos estatísticos e apresentação dos resultados, segundo esse recorte.

27. Apresente resultados mais completos e conclusivos. Defina o que são “idosos comunitários”.

28. No parágrafo “No nosso estudo as mulheres que faziam exercícios físicos não apresentaram sarcopenia, fato este que caracteriza o acesso à saúde como um comportamento predominantemente feminino, sendo necessário estratégias in loco para que os homens desde cedo, e não apenas quanto se tornarem idosos, busquem as atenção primária para cuidar da saúde e não para tratarem apenas de doenças” pode ter ocorrido uma inconsistência, pois na amostra do resultado formam incluídos pacientes com sarcopenia, não é possível que as mulheres não tenham apresentado sarcopenia.

29. Os resultados e as citações que foram aportadas na discussão não permitem fazer inferências sobre acesso à saúde.

30. Trecho “Estudos populacionais e mostraram uma prevalência menos de sarcopenia; vale lembrar que aqui estamos abordando pessoas idosas que muitas vezes apresentam vulnerabilidade quer seja no acesso aos serviços especializados quer seja por questões culturais sobre o processo de envelhecimento como se perder força e urina fosse normal para a idade.” Reveja a escrita buscando dar maior coerência entre as ideias. O que seria “mostraram uma prevalência menos de sarcopenia”? A ideia apresentada neste trecho da discussão pode causa uma confusão aos leitores, pois são ideias que não estão suportadas com os resultados. Nem todos os idosos apresentam vulnerabilidades, a população incluída teve acesso à APS e atenção especializada.

31. No trecho “É necessário avançar na organização do trabalho e, conforme levantado neste estudo com o aumento da prevalência da sarcopenia na atenção primária, o adequado dimensionamento das equipes, de condições de trabalho e processos robustos de formação e qualificação profissional são necessários uma vez que a sarcopenia não é um desafio para o futuro e sim para o agora”, o estudo não analisou se houve aumento ou redução da prevalência, não é possível fazer essa afirmação.

REVISÕES MENORES:

32. Reveja o texto do resultado, conforme orientações aos autores da revista. Evite termos genéricos como “evidenciando fatores de risco”. Faça indicações direta, exemplo: os fatores de risco para sarcopenia foi idade superior a 70 anos.

33. Padronize o uso dos conceitos, ou atenção primária ou atenção básica.

34. O texto da introdução precisa ser revisado para garantir clareza e aplicabilidade ao objetivo do estudo. No trecho “Nos últimos anos a uma discussão frequente surge sobre o modelo conceitual entre atenção primária à saúde e atenção básica, onde a primeira se refere porta de entrada preferencial que garante atenção oportuna aos serviços pertencentes ao Sistema Único de Saúde e o segundo modelo, idealizado e estruturado com enfoques para a promoção, prevenção, cuidado visando a saúde coletiva; enquanto predominam equívocos quanto ao seu significado e observam-se na prática um limbo entre o acesso aos serviços especializados e prevenção, que se caracteriza como um desafio principalmente às pessoas idosas no atual cenário de aumento populacional neste ciclo de vida(1,2).” Os últimos anos se refere a qual período? Qual a importância de destacar a disputa conceitual entre atenção básica versus atenção primária e o objeto de estudo proposto?

35. O manuscrito precisa ser revisado para garantir que esteja de acordo com as diretrizes do periódico. Revise a forma de apresentação dos valores numéricos, proporções precisam ser informadas com duas casas após a vírgula, p-valor com três casas decimais.

36. As citações devem ser feitas imediatamente após a passagem do texto em que é feita a citação, sem uso de espaço.

37. Assegure-se de que as citações estejam de acordo com o estilo da revista.” Veja as referências 7, 8, 13, 18 e 21.

38. Na tabela 2 suprimir as interrogações das variáveis de queda e incontinência urinária. O formato de pergunta foi utilizado na etapa de método, na tabela já é apresentação de uma variável afirmativa para risco.

